# Gut microbiota is involved in the alleviation of loperamide‐induced constipation by honey supplementation in mice

**DOI:** 10.1002/fsn3.1736

**Published:** 2020-06-27

**Authors:** Yuyuan Li, Shangqin Long, Qiaochu Liu, Hong Ma, Jianxin Li, Wei Xiaoqing, Jieli Yuan, Ming Li, Binbin Hou

**Affiliations:** ^1^ Advanced Institute for Medical Sciences Dalian Medical University Dalian China; ^2^ Department of Microecology College of Basic Medical Science Dalian Medical University Dalian China; ^3^ The Core Laboratory of Medical Molecular Biology of Liaoning Province Dalian Medical University Dalian China; ^4^ The Second Hospital of Dalian Medical University Dalian China

**Keywords:** constipation, honey, intestinal microbiota

## Abstract

Constipation is one of the most common functional gastrointestinal disorders accompanied with intestinal dysbiosis. Laxatives for constipation usually have side effects. Bee honey is a natural food with unique composition, antimicrobial properties, and bifidogenic effect. In order to assess whether honey can ameliorate loperamide‐induced constipation in BALB/c mice through the alteration of the gut microbiota, the present study was undertaken. Mice were given Jarrah honey (7.5 g/kg body weight) by gavage once per day for 5 days. Fecal water content, intestinal transit rate together with the colon concentrations of substance P (SP), vasoactive intestinal peptide (VIP), and serotonin (5‐hydroxytryptamine; 5‐HT) were evaluated. Furthermore, we determined the effect of honey treatment on gut microbiota in mice using stool genomic 16S rRNA sequencing. As a result, honey showed an obvious improvement in fecal water content and alleviated constipation by modulating the microbial composition of the microbiota, and this was highly associated with a proportional decrease in gut *Desulfovibrio*. In addition, we found that the colon level of neurotransmitters SP and VIP was significantly related to microbial variations. Our results indicate that gut microbiota is involved in the alleviation of loperamide‐induced constipation by honey supplementation in mice, and it could be considered as an evaluating parameter in constipation therapy strategies.

## Graphical abstract

This study employed 16S rRNA gene sequencing to investigate the composition and function of the gut–microbiome in loperamide‐induced constipation mice model treated with honey. Microbiological analysis revealed that honey administration can manipulate intestinal dysbiosis by reducing the number of harmful bacteria in the intestines of constipated mice.

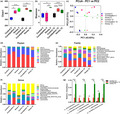

**Abbreviations**
5‐HT: 5‐hydroxytryptamine; serotonin
CCA: canonical correlation analysis
GI: gastrointestinal
LDA: linear discriminant analysis
LEfSe: linear discriminant analysis effect size
OTUs: operational taxonomic units
PCoA: principal coordinate analysis
SCFA: short‐chain fatty acids
SP: substance P
VIP: vasoactive intestinal peptide

## 1 INTRODUCTION

Constipation is a globally prevalent functional gastrointestinal (GI) disorder and public health problem, characterized by difficult and infrequent defecation, dry and hard stool, and prolonged the colonic and rectal emptying time (Camilleri et al., 2017). The prevalence of constipation ranged from approximately 9% to more than 20%, especially in the elderly (Bassotti, 2013). Moreover, the incidence of constipation showed a gradual upward trend due to the adjustment of diet structure and the acceleration of the pace of life in recent years. In addition, constipation may be associated with increased risk of many related diseases such as atherosclerosis (Sumida et al., 2019), Parkinson's disease (Barichella et al., 2016), and colorectal cancer (Abraham & Taylor, 2017). Consequently, it is very necessary to prevent and treat constipation.

Growing evidences showed that constipation was associated with intestinal dysbiosis. Disruption of intestinal microbiota balance is characterized by a relative decrease in probiotic bacteria and an increase in potentially pathogenic bacteria, which may further affect the intestinal immune function and barrier function and influence health (Henao‐Mejia, Elinav, Thaiss, Licona‐Limon, & Flavell, 2013). Several clinical studies have investigated gut microbial communities in constipation. Specifically, studies have found that the relative levels of methanogenic *archaea* (Kang et al., 2015) and *clostridia* (Zoppi et al., 1998) increase in patients with constipation. Khalif, Quigley, Konovitch, and Maximova (2005) reported that the abundances of *Bifidobacteria* and *Lactobacillus* in stool samples were significantly decreased in adult patients with constipation. Moreover, some animal studies have also found that intestinal dysbiosis could conduce to the development of constipation (Liu et al., 2018; Takayama, Takahara, Tab uchi, & Okamura, 2019). Thus, altering the intestinal microbiota in the gut may contribute to the treatment of constipation.

So far, several drugs have been used to treat this disorder. In most instances, chemical drugs (laxatives) act as bulk agents, stool softeners, stimulants, and prokinetic agents (Kim et al., 2018). However, most of these drugs had adverse side effects, such as myocardial infarction, artery contraction, coronary spasms, and colorectal neoplasm (Busti, Murillo, & Cryer, 2004; Kim, 2013; Lembo & Camilleri, 2003; Siegers, von Hertzberg‐Lottin, Otte, & Schneider, 1993). Honey is a natural substance produced by bees from nectar, which has been used not only as a great nutrient, but also as a medicine since many centuries ago (Zumla & Lulat, 1989). It has been observed that honey can be used to overcome wounds, diabetes mellitus, cancer, asthma, and also cardiovascular, neurological, and gastrointestinal diseases (Samarghandian, Farkhondeh, & Samini, 2017). It contains oligosaccharides or bifidogenic factor that can serve as prebiotics (Vahdat, Jamshidi, Nasrollahzadeh, Amiri, & Teymourian, 2018). Evidence indicates that honey can be used to overcome gastrointestinal problems, such as ethanol or NSAID‐induced gastric mucosal injury in the rat (Gharzouli, Amira, Gharzouli, & Khennouf, 2002). It has been revealed that honey supplementation was associated with changes in colonic microbiota, especially increases of *Lactobacillus* and *Bifidobacterium*, of preterm infants (Aly et al., 2017). No study has indicated the effects of honey supplementation on the intestinal microbiota and constipation. The purpose of this study was to determine whether honey could relieve constipation induced by loperamide in mice and whether it was through the manipulation of gut microbiota dysbiosis.

In this study, honey derived from *Eucalyptus marginata* (jarrah), which was the most potent antibacterial honey yet reported (Irish, Blair, & Carter, 2011), was administered to mice with loperamide‐induced constipation. Effects of Jarrah honey on small intestine transit and fecal water content, as well as the changing of intestinal microbial structures in mice, were evaluated using next‐generation sequencing. This study will provide a new perspective on honey treatment for constipation.

## 2 MATERIALS AND METHODS

### 2.1 Ethics statement

The animal experiments was reviewed and approved by Dalian Medical University Institutional Animal Care and Use Committee in accordance with the laboratory's animal ethics guidelines (SYXK [Liao] 2014–0002).

### 2.2 Experimental animals

Seven‐week‐old male BALB/c mice weighing 18–22 g (*n* = 30) were obtained from the specific pathogen free animal center of Dalian Medical University, China. All mice were maintained under standard conditions at a room temperature of 25 ± 2°C and humidity of 50% ± 5% with a 12‐hr light–dark cycle. They were fed normal mouse‐chow diet with ad libitum access to water and fasted for 24 hr prior to all experiments.

### 2.3 Induction of constipation and experimental design

**FIGURE 1:**
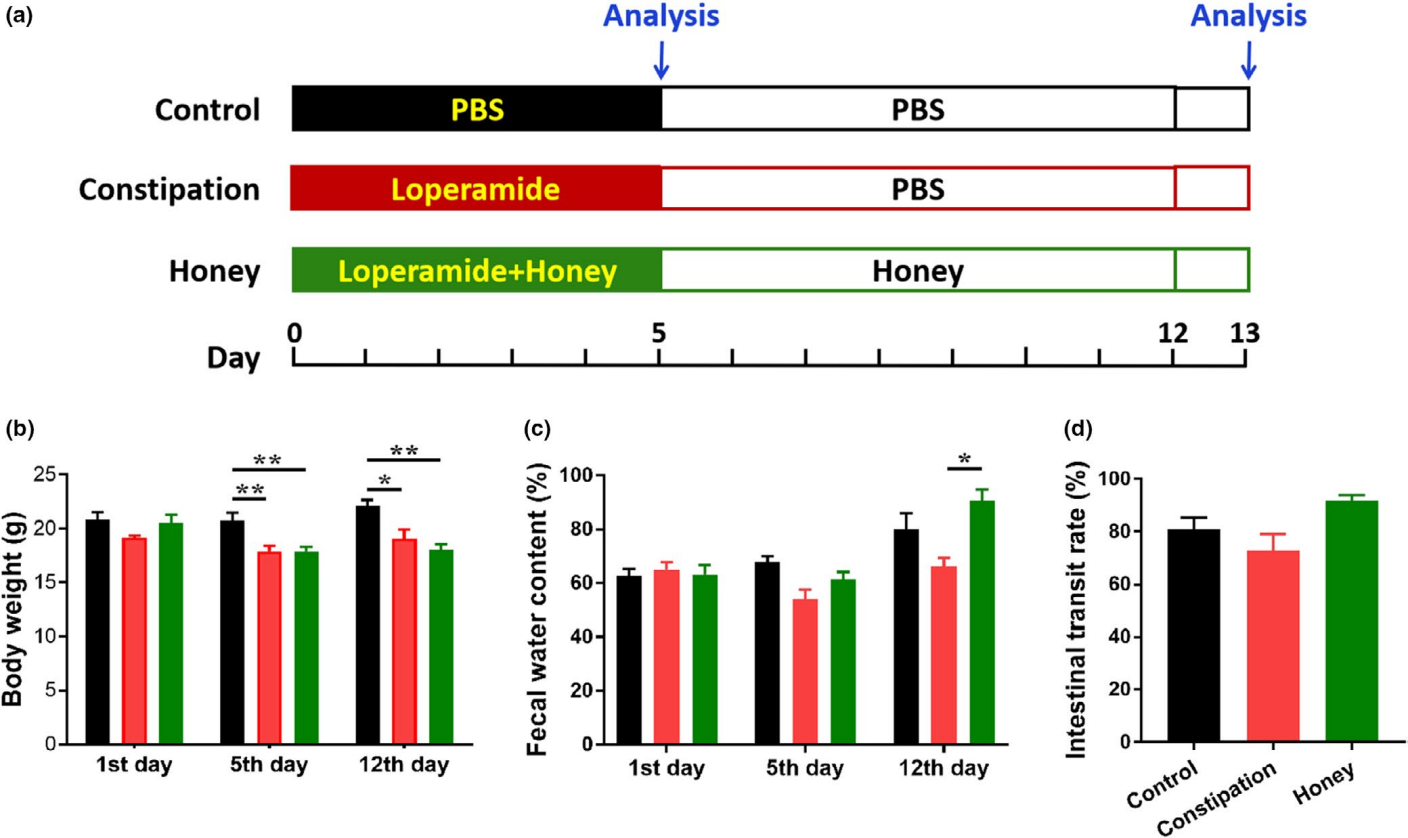
Effects of honey on the physical features in loperamide‐induced constipated mice. (a) Study design. Except for control mice (*n* = 10), mice were oral gavaged with loperamide (5 mg/kg) once each day from day 1 to day 5 to induce constipation. Additionally, mice in the honey group (*n* = 10) were also intragastrically administered honey (7.5 g/kg) once each day from day 1 to day 12. Mice in the control and constipation groups were given 0.2 ml PBS as vehicle. (b) The body weight, (c) fecal water content, and (D) intestinal transit rate of mice were measured in each group at days 5 and 12. *p < .05; **p < .01, data represent mean ± *SEM* of ten mice in each group

As shown in Figure 1a and Table 1, mice were randomized into three groups (*n* = 10 for each group): the control group, the constipation group, and the honey group. After one‐week adaptive feeding, loperamide (Sigma) was used to induce slow‐transit constipation in mice. The constipation and honey groups were given loperamide at a dose of 5 mg/kg body weight, once per day (18:00) via oral gavage from day 1 to day 5. Mice in the honey group were given Jarrah honey (Elixir, TA (total activity) 45+ Eucalyptus marginata, purchased from Dalian aoxinbaiying International Trade Co., Ltd) suspended at a dose of 7.5 g/kg body weight in 0.2 ml PBS once a day (9:00) for 12 days, while the constipation group was administrated 0.2 ml of PBS as vehicle. The control group was given PBS by gavage twice a day (9:00 and 18:00) from day 1 to day 5, and once a day (9:00) from day 6 to day 12. Mortality, body weight, and fecal output were recorded daily. At 24 hr after final treatment, all animals were anesthetized before sacrifice. The gastrointestinal tract was removed and emptied of contents. The transverse colon, blood, and feces of mice were collected and immediately placed at −80°C until detection.

**TABLE 1:** Specifics of the animal experiment

Group	Treatment		
	1–5 days		6–12 days
	9:00	18:00	9:00
Control	PBS	PBS	PBS
Constipation	PBS	loperamide	PBS
Honey	honey	loperamide	honey

### 2.4 Detection of fecal water content

Fecal samples were collected at 10:00 on day 5 and day 12, and dried in an oven at 60°C for 24 hr. The water content of feces was calculated as follows: fecal water content (%) = (feces weight before dried ‐ feces weight after dried)/ feces weight before dried × 100.

### 2.5 Analysis of intestinal transit rate

Intestinal transit ratio was conducted according to previously reported protocols (Yin et al., 2018); briefly, after 12 days of treatment, all mice were fasted for 24 hr but were allowed free access to water. After that, mice from each group were fed 1 ml of active carbon powder solution (3.0 g carbon powder suspended in 50.0 ml 0.5% CMC‐NA solution, Kermel Chemical Reagent Co., Ltd.). Thirty minutes after administration of the carbon powder solution, the mice were sacrificed and dissected to collect the small intestinal segments between pylorus and ileocaecal junction. The active carbon was used as an indicator. The distance from pylorus to ileocaecal junction, as the total small intestine length, and the active carbon transfer length were measured. The following equation estimates the intestinal transit rate (%): distance travelled by the active carbon/ total small intestine length × 100%.

### 2.6 ELISA analysis

At the time of sacrifice, the transverse colons of mice were excised, freed of adherent adipose tissue, and stored at −80°C for subsequent analysis. Appropriate amount of colon tissue was homogenized and centrifuged to get the supernatant. The concentrations of substance P (SP), vasoactive intestinal peptide (VIP), and serotonin (5‐hydroxytryptamine; 5‐HT) were determined in supernatant of colon tissue homogenates using corresponding detection kits (Shanghai Langton biotechnology Co. Ltd), according to the manufacturer's instructions.

### 2.7 16S rDNA sequencing and analysis

Fecal DNA was extracted from stool samples in the morning on day 5 and day 12 of the three groups using E.Z.N.A.^®^ Stool DNA Kit (Omega Bio‐Tek) following to the manufacturer's guidelines. The V3‐V4 region of 16S ribosomal DNA from metagenomic DNA in mice feces was amplified with universal primers (515F, 806R). The products were excised from 1.5% (w/v) agarose gels and purified using the QIAquick Gel Extraction kit (Qiagen, Germany) according to the manufacturer's instructions. Sequences were detected on an Illumina HiSeq 2000 platform by Novogene using a method described previously (Deng et al., 2018). Operational taxonomic units (OTUs) present in 50% or more of the fecal samples were identified as core OTUs. Based on the results of OTUs, alpha diversity and beta diversity were analyzed subsequently. Alpha diversity was evaluated by Shannon–Wiener biodiversity index (Shannon's index). Community richness was evaluated by Chao1. Furthermore, beta diversity was investigated by visual assessment using principal coordinates analyses (PCoA) to analyze differences of samples. The OTU relative abundance values were analyzed using the linear discriminant analysis effect size (LEfSe) algorithm to identify the bacterial taxa that differentially represented between groups at different taxonomic levels. A linear discriminant analysis (LDA) was performed to assess the effect size of each differentially abundant taxon.

### 2.8 Statistical analysis

Statistical analysis was performed by SPSS 19.0 system software (SPSS Inc.). All the experiments were performed in triplicates, and data were presented as arithmetic mean ± *SEM*. The data sets involved in more than two groups were assessed using one‐way ANOVA followed by Tukey's test with the assistance of GraphPad Prism Program (Version 7.04; GraphPad Software Inc.). In the analyses of the significance for PCoA (beta diversity), we used a permutational multivariate analysis of variance (PERMANOVA) approach (“adonis” function in the R package “vegan”) that adjusts for potential confounding covariates. Community comparison was evaluated using a Student's *t* test. A *p*‐value of less than 0.05 was considered as statistically significant.

## 3 RESULTS

### 3.1 Effects of the honey on body weight, fecal water content, and intestinal transit rate of constipated mice

A diagram illustrating our study design is shown in Figure 1a. There was a significant decrease in body weights in constipated mice by day 5 (from 20.71 ± 0.80 to 17.91 ± 0.50, *p* = .0084) and day 12 (from 22.086 ± 0.605 to 19.086 ± 0.833, *p* = .0111; Figure 1b). However, there was no significant difference in body weight between the honey group and the constipation group, which implied that honey can not effectively restore weight to normal levels. The water content of feces was calculated and shown in Figure 1c. After treatment with loperamide for 5 days, the fecal water content in the constipation group showed a decrease trend compared with the control group on day 5 (*p* = .0939) and day 12 (*p* = .2913). After 12 days of treatment with honey, a significant improvement in fecal water content was observed in the treatment group when compared to the constipation group on day 12 (*p* = .0263; Figure 1c). As shown in Figure 1d, loperamide decreased the intestinal transit rate tendentiously (from 80.89 ± 4.46% to 72.65 ± 6.55%, *p* = .4739), and honey had the effect in improving the rate of intestinal transit (up to 91.72 ± 2.19%, *p* = .1111).

### 3.2 Effects of the honey on SP, VIP, and 5‐HT levels in the mice colon

**FIGURE 2:**
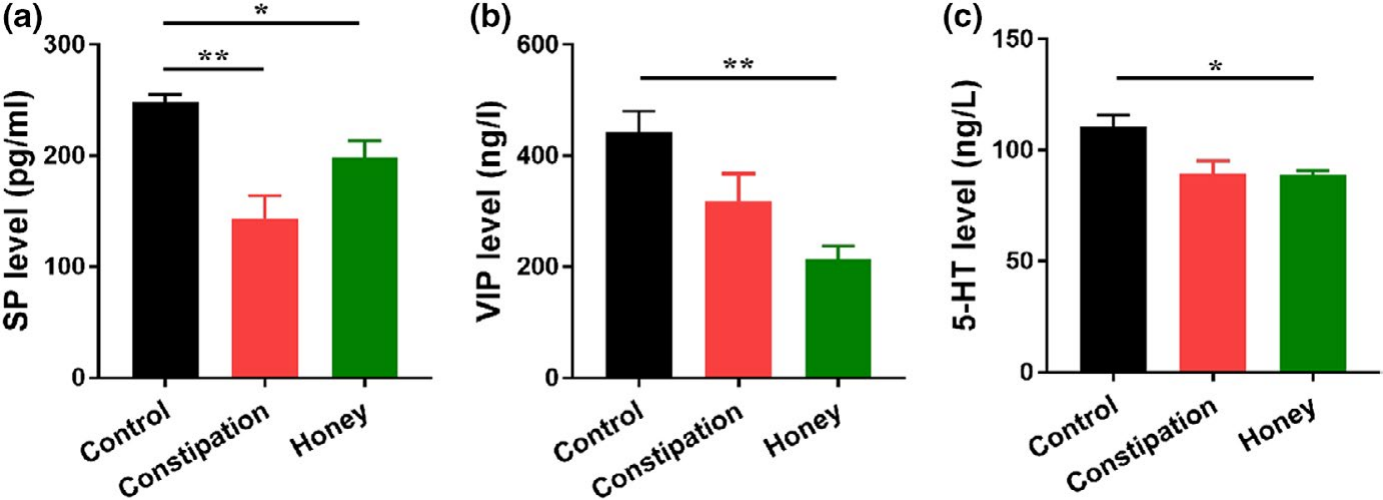
Effect of honey on brain–gut peptide in the colonic tissue of constipation mice. (a) Substance P (SP), (b) vasoactive intestinal peptide (VIP), and (c serotonin (5‐HT) levels in colonic tissues of mice from different experimental groups (*n* = 10) were analyzed by ELISA. *p < .05; **p < .01, data represent mean ± *SEM* of ten mice in each group

A number of major gastrointestinal hormones associated with motilin were altered in constipated mice. As shown in Figure 2, the colon level of SP in mice with constipation was lower than those in healthy individuals (*p* = .006), while the VIP (*p* = .3352) and 5‐HT (*p* = .1654) levels were not changed. Honey treatment leads to a decreasing trend in the contents of inhibitory neurotransmitter VIP and 5‐HT, and an increasing trend in the expression of excitatory neurotransmitter SP (all *p* > .05).

### 3.3 Honey re‐established the equilibrium of intestinal microecosystem

**FIGURE 3:**
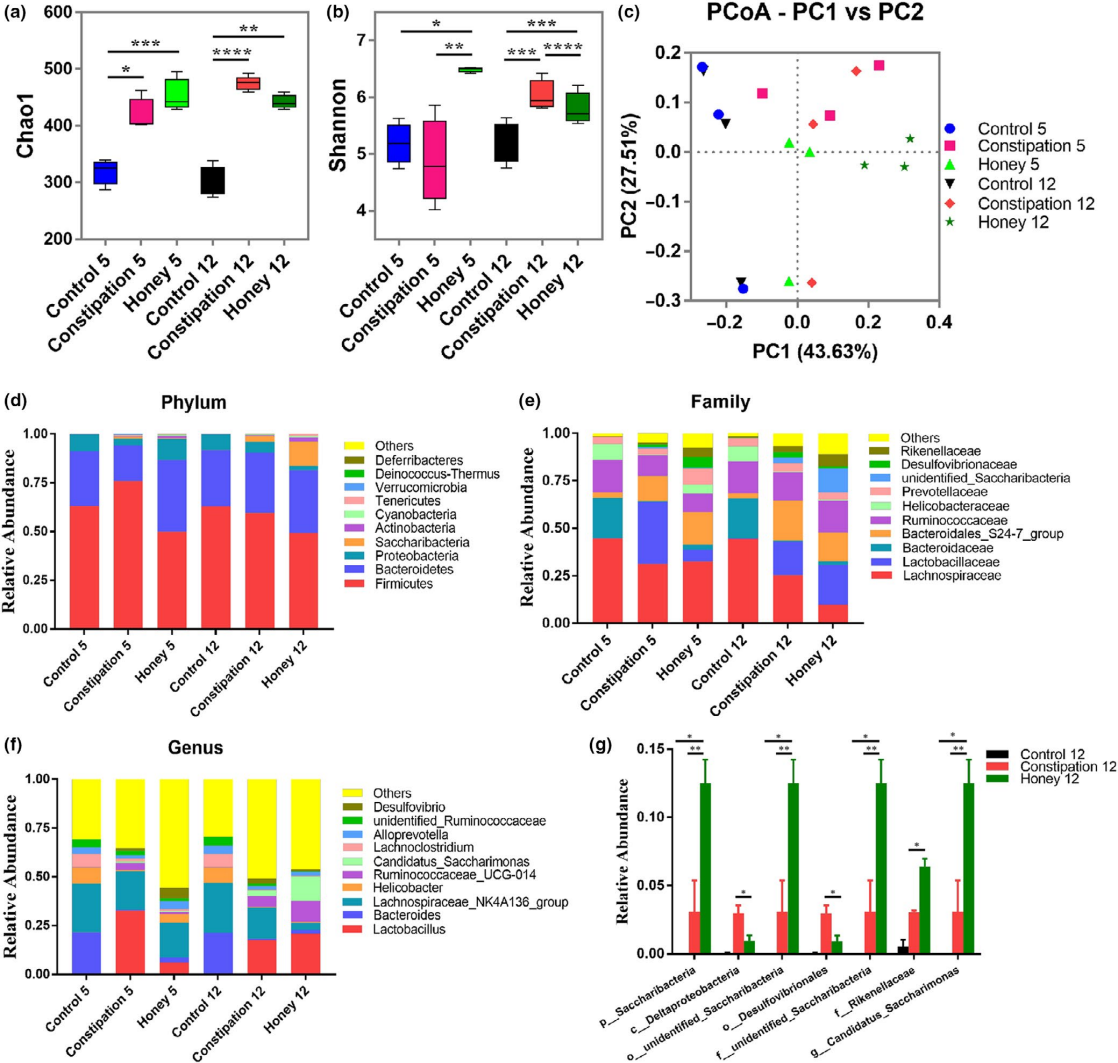
Structural comparison of fecal microbiota among three groups. (a) Comparison of the Chao 1 index of different groups. (b) The Shannon index was used to estimate diversity of the fecal microbiota among the three groups. (c) Plots shown were generated using the weighted UniFrac‐based PCoA. PC1 and PC2 account for 71.14% of the variation. (d–f) The relative abundance of microbial community in different mice groups at phylum, family, and genus levels among the three groups. (g) The relative abundance of bacterial groups on day 12 between groups tested by means of one‐way ANOVA followed by Tukey's test. *p < .05; **p < .01; ***p < .001; ****p < .0001

The modulatory effect of honey on the intestinal microbiota was investigated by sequencing of the V3‐V4 region of the 16S rRNA gene of the predominant bacteria in mice. As shown in Figure 3a, the Chao1 and Shannon indexes were used to evaluate the species richness and diversity within each microbiome sample (alpha diversity). The Shannon index of mouse gut microbiota was found increased significantly by loperamide treatment on day 5 (*p* = .0132) and day 12 (*p* < .0001). However, the Chao1 index in the honey group had no change compared with the constipation group (*p* = .2540 and 0.0555 on day 5 and day 12, respectively). As depicted in Figure 3b, the value of the Shannon index for the constipation group was significantly higher compared with the control group (*p* = .0003) while the honey group was significantly lower compared with the constipation group (*p* < .0001). This suggests that the microbial diversity was greatly improved by honey treatment on day 12. Additionally, the PCoA was used to visually assess the similarities of the ecological complexities among all of the microbiome samples (beta diversity). Each point represented the microbial community of one sample. According to the results of the weighted UniFrac PCoA (Figure 3c), the honey group was significantly different from the other two groups (all *p* = .0014), suggesting that honey can change the composition of intestinal microbiota on day 12. A significantly decreased abundance of *Proteobacteria* (phylum), *Bacteroidales* (order), *Helicobacteraceae* (family), and *Lachnoclostridium* (genus) was observed in loperamide treated group compared to the control group (Figure 3d–f). In addition, an elevated abundance of *Lactobacillales* (order), *Desulfovibrionales* (order), *Lactobacillaceae* (family), and *Desulfovibrionaceae* (family) was found in constipated mice (Figure 3d–f). We also found that the ratio of *Firmicutes* to *Bacteroidetes* (F/B), a widely used marker of gut dysbiosis (Turnbaugh et al., 2009), was lower in honey group when compared to constipation group on day 5 (Figure S1a). And the Bacteroides level was higher in honey group than constipation group on day 5 (Figure S1b). It was obvious that honey treatment had recovered the altered microbial structure in mice with loperamide‐induced constipation, particular for the strain of *Desulfovibrinonales* (Figure 3g).

### 3.4 The differentiated microbial taxa detected in each group

**FIGURE 4:**
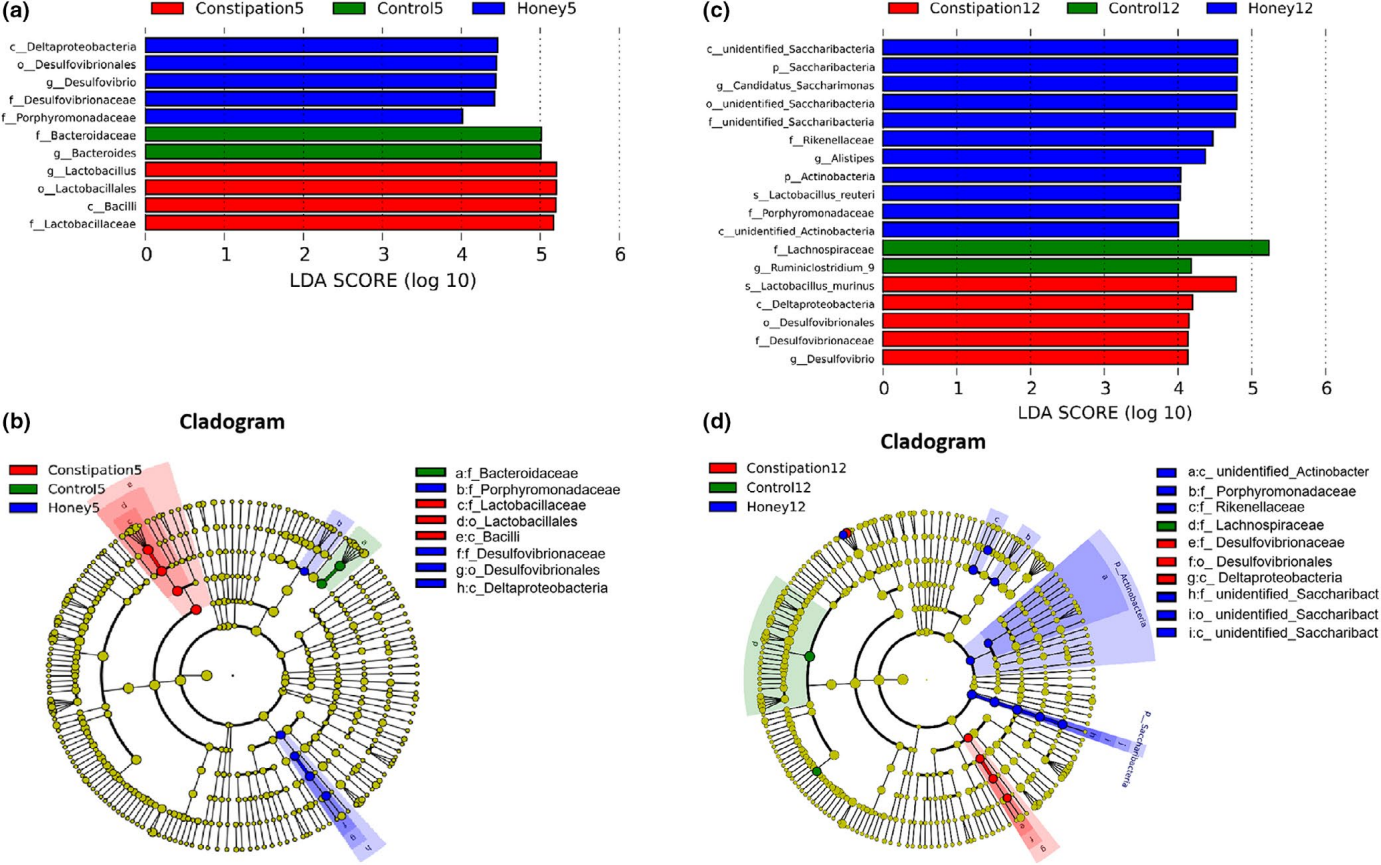
LEfSe analysis of intestinal microbiota among different mice groups. (a, c) LEfSe identified the most differentially abundant bacterial taxons among groups on days 5 and 12. Group‐specific enriched taxa are indicated with a positive LDA score bar with different colors. Only taxa meeting an LDA significant threshold >4 are shown. (b, d) Taxonomic cladogram obtained from LEfSe analysis of 16S rDNA sequences on day 5 and 12. The brightness of each dot is proportional to its effect size

The LEfSe approach was used to detect the bacterial groups that significantly differed in one mice group when compared with others. The results were shown in Figure 4, as displayed by LDA scoring plot (LDA > 4), *Bacteroidaceae* (5 days) and *Lachnospiraceae* (12 days) were most abundant microbial groups found in the control mice; *lactobacillaceae* (5 days) and *Deslfovibrionaceae* (12 days) were found most abundant in the constipated mice; and *Deslfovibrionaceae* (5 days) and *Saccharimonas* (12 days) were most abundant in the honey group, and these microbial groups are dominant phylotypes that contributed to the differences among three groups (Figure 4).

### 3.5 The abundance of specific bacterial groups in mice showed correlation with levels of neurotransmitters

**FIGURE 5:**
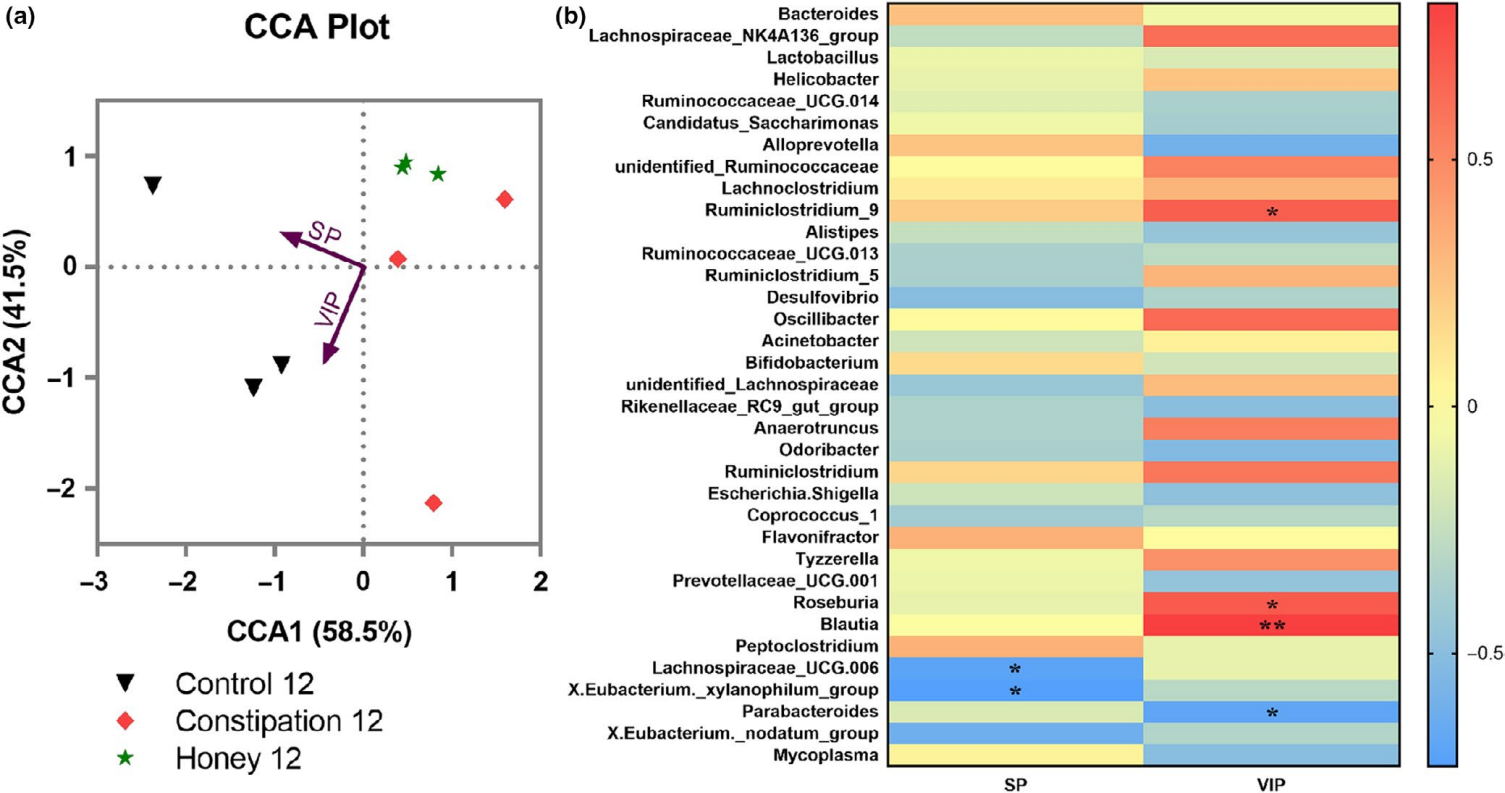
The correlation between intestinal bacterial groups and neurotransmitters. (a) Canonical correlation analysis (CCA) analysis. Arrows represent the variables substance P (SP) and vasoactive intestinal peptide (VIP) and indicate the direction and magnitude of the variables associated with bacterial community structure. (b) The correlation between the relative abundance of different microbial groups at genus level and the levels of SP and VIP was tested by the Spearman correlation method. The positive correlation was displayed as correlation value >0, and the negative correlation was displayed as correlation value <0; statistically significant correlation was displayed as *p < .05; **p < .01

Mantel tests and the canonical correlation analysis (CCA) indicated that the colon level of neurotransmitters SP and VIP was the major factors contributing to the differences between the bacterial communities and environmental factors (*p* = .0045, Figure 5a). We also analyzed the Spearman correlation between intestinal bacterial groups and the colon level of neurotransmitters SP and VIP (Figure 5b). It was found that bacteria belonging to the genus of *Lachnospiracaeae_UCG.006* (*p* = .0358) and *X.Eubacterium._xylanophilum_group* (*p* = .0262) were negatively correlated with the upregulation of SP, while *Parabacteroides* (*p* = .0424) were negatively correlated with the upregulation of VIP in mouse colon. In contrast, bacterial groups, such as the genus of *Ruminiclostridium_9* (*p* = .0424), *Roseburia* (*p* = .0358), and *Blautia* (*p* = .0072), were positively correlated with the increase of VIP in mouse colon.

## 4 DISCUSSION

Constipation is a prevalent functional gastrointestinal disease worldwide and can lead to serious damage of quality of life, and also bring about huge socioeconomic burdens on both individuals and national health insurance (Quigley & Spiller, 2016; Wintola, Sunmonu, & Afolayan, 2010). Natural products and medicinal foods are attracting more and more attention nowadays because of their laxative and no side effects on constipation (Irish et al., 2011; Kakino et al., 2010). Honey is a natural product of flower nectar and the upper aero‐digestive tract of the honey bee, which has been used both as food and medicine since ancient times (Eteraf‐Oskouei & Najafi, 2013). The most remarkable discovery was antimicrobial properties and bifidogenic effect of honey that has been mentioned in numerous studies (Chow, 2002; Ezz El‐Arab, Girgis, Hegazy, & Abd El‐Khalek, 2006). Thus, we investigated the ameliorative effects of the honey on loperamide‐induced constipated mice by using honey derived from *Eucalyptus marginata* (jarrah), which was the most potent antibacterial honey yet reported (Henao‐Mejia et al., 2013). The results of this study suggested that honey can improve the symptoms of constipation by elevating fecal water content and intestinal transit rate in loperamide‐induced constipation model.

Analysis of body weight in this study found that significantly lower body weight was observed in the constipation group compared with the control group (Figure 1). This finding is consistent with the report of Liu, Lin, Lin et al. (2019) and Liu, Lin, Sun et al. (2019), which demonstrated that constipation caused the decrease of the body weight and the food intake. The honey group still saw the lowest body weight on day 12, yet it witnessed the highest fecal water content and intestinal transit rate. The results showed that loperamide‐induced chronic transit constipation treated with honey can improve the fecal water content and intestinal transit rate, but not recover the body weight in a very short time.

The gut microbiota plays various important functions for the host health, such as structural, metabolic, and protective roles as well as a direct action on the gut mucosa, the enteric nervous system, and far beyond the local GI compartment (Grenham, Clarke, Cryan, & Dinan, 2011; Neish, 2014; Trompette et al., 2014). The alterations of gut microbiota may contribute to constipation and constipation‐related symptoms, which have recently attracted considerable interest among gastrointestinal researchers (Gerritsen, Smidt, Rijkers, & de Vos, 2011; Khalif et al., 2005; Parthasarathy et al., 2016). To decipher the underlying mechanism of honey in the treatment of constipation, we investigated the influence of honey on intestinal microbiota. In the present study, alpha‐diversity analysis demonstrated that the diversity and richness of the bacterial communities in constipation group were higher than that in control group (Figure 3). Some previous studies have indicated that the control group showed higher bacterial diversity and richness than constipation group, demonstrating that more different communities may correspond to healthier ecosystems (Ren, Liu, Gamallat, Zhang, & Xin, 2017). Moreover, other studies reported that bacterial diversity and richness were similar between two groups (Wang et al., 2017). It was noteworthy that some previous studies demonstrated that increased alpha diversity was significantly associated with longer colonic passage (Müller, Hermes, Canfora, Holst, et al., 2020; Müller, Hermes, Canfora, Smidt, et al., 2020), reflecting an adaption to a changing ecosystem (i.e., depletion of nutrients, switch from microbial saccharolytic to proteolytic fermentation, microbial competition, decreased water availability; Falony, Vieira‐Silva, & Raes, 2018). Therefore, the conclusion among studies still remains controversial and further research is required. Constipation can induce the overgrowth of many harmful bacteria, such as *Firmicutes*, and reduce the abundance of beneficial bacteria, such as *Bacteroides*. Some species from *Firmicutes* cause inflammation in various organs and tissues, which will show dysfunction, and the intestinal tract can directly reflect dysfunction due to intestinal microecology (Yi et al., 2019). The *Firmicutes* phylum has also been found positively association with the pathways involved in hydrogen production and methanogenesis, which may potentially regulate the constipation development (Kang et al., 2015; Kurokawa et al., 2018; Mancabelli et al., 2017). Moreover, previous studies have suggested that an increased ratio of the major phyla *Firmicutes* to *Bacteroidetes* can promote the development of obesity (Chang et al., 2015), which has the significantly association with constipation (Pourhoseingholi et al., 2009). Regulating the proportions of these microorganisms in the intestines can improve the intestinal environment, thereby alleviating constipation.

As shown in Figure 3, one of the most significant findings was that the level of *Desulfovibrionales* (including *Desulfovibrio*) was lower in the honey group compared with the constipation group. And *Desulfovibrionales* (12 day) were found most abundant in the constipated mice (Figure 4). Of note, prior literatures have well established that *Desulfovibrionales* is harmful for constipation (Cao et al., 2017; Liu, Lin, Lin et al., 2019); Liu, Lin, Sun et al.,^2019^). Bacteria of the genus *Desulfovibrio* is a gram‐negative sulfate‐reducing bacteria (SRB), which accounts for 66% of all SRB in the human colon and can perform anaerobic respiration utilizing lactic acid, pyruvate, ethanol, and fatty acids to reduce sulfate into hydrogen sulfide (H_2_S) (Muyzer & Stams, 2008). H_2_S may be toxic to intestinal epithelial cell integrity and proliferation (Linden, 2014), blocking the butyrate oxidation pathways in colon cells and inducing apoptosis and chronic inflammation (Hulin et al., 2002). It was also known to inhibit gastrointestinal motor function (Jimenez, Gil, Martinez‐Cutillas, Mane, & Gallego, 2017). In human studies, it has been reported that the level of *Desulfovibrio* in patients with ulcerative colitis is higher compared with healthy subjects (Coutinho et al., 2017; Earley et al., 2015; Jia et al., 2012). It has also been shown that there were significantly higher levels of *Desulfovibrio* ssp. in the constipated patients (Jalanka et al., 2019). Our results confirm the association of *Desulfovibrio* with constipation which may be followed up by further research on the relationship between this organism and GI health. In addition, the abundance of some bacterial groups presented a significant difference in gut of the constipation group of mice. We found that the relative abundance of *Proteobacteria* is significantly decreased at phylum level in constipated mice. Similarly, Guo et al. (2020) reported the significant decrease of *Proteobacteria* in constipated patients. The research of Li et al. (2019) also showed that the abundance of *Proteobacteria* was lower in the constipation‐predominant intestine tumor group than the control group. Additionally, the *Desulfovibrionales* are an order of *Proteobacteria*, which may be the main reason for the difference of *Proteobacteria* levels among the groups. As shown in Figures 3 and 4, the bacterial phylum *Saccharibacteria* (formerly known as phylum TM7) was abundant in the honey group. *Saccharibacteria* are ubiquitous members of the human microbiome and are detected in various human body sites, such as the gastrointestinal tract, skin, and female genital tract (Bor, Bedree, Shi, McLean, & He, 2019). However, to our knowledge, few studies have investigated the relationship between *Saccharibacteria* and constipation, which needs further research. In addition, many *Lactobacilli* have been demonstrated to play beneficial roles in constipation, such as promoting peristalsis and defecation (Naseer, Poola, Uraz, & Tahan, 2020; Yi et al., 2019). In this study, *Lactobacillus,* especially the species of *Lactobacillus murinus* (*L. murinus*), were found most abundant in the constipated mice (Figure 4), which aroused our attention. Hayashi et al. (2017) reported that the overgrowth of *L. murinus* impaired gut metabolic function, which suggested that a detail study focus on species level of *Lactobacilli* will provide more information about the effects of this microbial group in gut. Nevertheless, to the best of our knowledge, there have been no reports on the role of *L. murinus* in constipation.

Neurotransmitter receptors also play essential roles in intestinal muscle movement, and some neurotransmitters have been identified to contribute to the dynamic function of GIT including inhibitory factors 5‐HT and VIP, and excitatory factor SP (Mao et al., 2017). 5‐HT is a common neurotransmitter in the brain–gut axis, which expression in colonic mucosa was reported to be decreased in chronic constipation patients, suggesting that 5‐HT may crucial for the treatment of constipation (Coates et al., 2004). In this study, we found that the mice in constipation group showed comparatively low colon level of 5‐HT (Figure 2). However, honey treatment presented no effect on the change of 5‐HT. SP is an excitatory transmitter of GI motor neurons. It strongly promotes smooth muscle contraction, stimulates intestinal water and electrolyte secretion, and promotes GI peristalsis (Yi et al., 2019). VIP is a 28 amino neuropeptide, which is widely located in neurons of the central and peripheral nervous systems (Augustin & Lutz, 1991). Although it is a type of noncholinergic inhibitory neuropeptide, it has been proved to mediate promotion of colon, thus stimulating intestinal peristalsis. Abnormalities of the neurotransmitter SP and VIP may contribute to the incidence of constipation (Moriya et al., 2010). Deficiency of SP can impede intestinal peristalsis (Xiong et al., 2014), while reduction of VIP concentration can inhibit the effective promotion of colon and lead to constipation (Giancola et al., 2017). In our study, the colon levels of SP and VIP in mice with constipation was lower than those in healthy individuals, and honey administration had no effects on the expression of the neurotransmitter SP and VIP. Based on these results, we hypothesized that the amelioration effect of honey on constipation of mice was mainly through the increasing of fecal water content, but not by stimulating intestinal peristalsis, which was consistent with the result of Figure 1.

Both the CCA and the Spearman correlation analyses illustrated that the colon level of neurotransmitters SP and VIP was significantly related to microbial variations (Figure 5). We identified that abundances of key butyrate‐producing taxa (*Lachnospiraceae* and *Eubacterium*) were negatively correlated with the upregulation of SP in mouse colon. Butyrate is one of the most significant metabolites of the gut microbiome and regulates the role of the microbiome–gut–brain axis (Liu, Lin, Lin et al., 2019); Liu, Lin, Sun et al.,^2019^). We also observed that abundance of taxa *Roseburia* was positively correlated with the increase of VIP in mouse colon. According to a previous study (Duncan, Hold, Barcenilla, Stewart, & Flint, 2002), *Roseburia* can produce short‐chain fatty acids (SCFA) that provide energy for the intestinal mucosa cells and stimulate differentiation and proliferation of cells in the colon. Moreover, SCFA can also lower the pH of the intestines, inhibit the growth of harmful bacteria in the gut, and improve the intestinal function. It has also been reported that *Roseburia* was correlated with faster colonic transit (Parthasarathy et al., 2016).

## 5 CONCLUSIONS

In conclusion, this study was conducted to investigate the composition and function of the gut microbiome in loperamide‐induced constipation mice model treated with honey. Microbiological analysis demonstrated that honey administration can manipulate intestinal dysbiosis by suppressing harmful bacteria in the intestines of constipated mice. However, the present study has been limited on the sample size in the Illumina HiSeq sequencing. Further studies on a larger number of samples should be conducted. Overall, this study extends our current knowledge of the potential therapeutic role of honey in constipation and offers insight into the effects of honey in loperamide‐induced constipation model based on microbiology.

## CONFLICTS OF INTEREST

The authors have declared no conflict of interest.

## AUTHOR CONTRIBUTION

YL analyzed the data and wrote the manuscript; SL, QL, and HM performed the experiments; JL, XW, and JY analyzed and interpreted the data; ML obtained the funding, designed the research, and revised the manuscript; and BH designed the research and revised the manuscript. All authors read and approved the final manuscript.

## ETHICAL APPROVAL

All experimental protocols in the current study were approved by the Animal Ethical Committee of Dalian Medical University.

## Supporting information

Fig S1
