# Supplementary figures and images for: Gut microbiota is involved in the alleviation of loperamide‐induced constipation by honey supplementation in mice
Source: Food Sci Nutr. 2020 Jun 27;8(8):4388–98. doi: 10.1002/fsn3.1736 (PMC7455974; doi:10.1002/fsn3.1736)

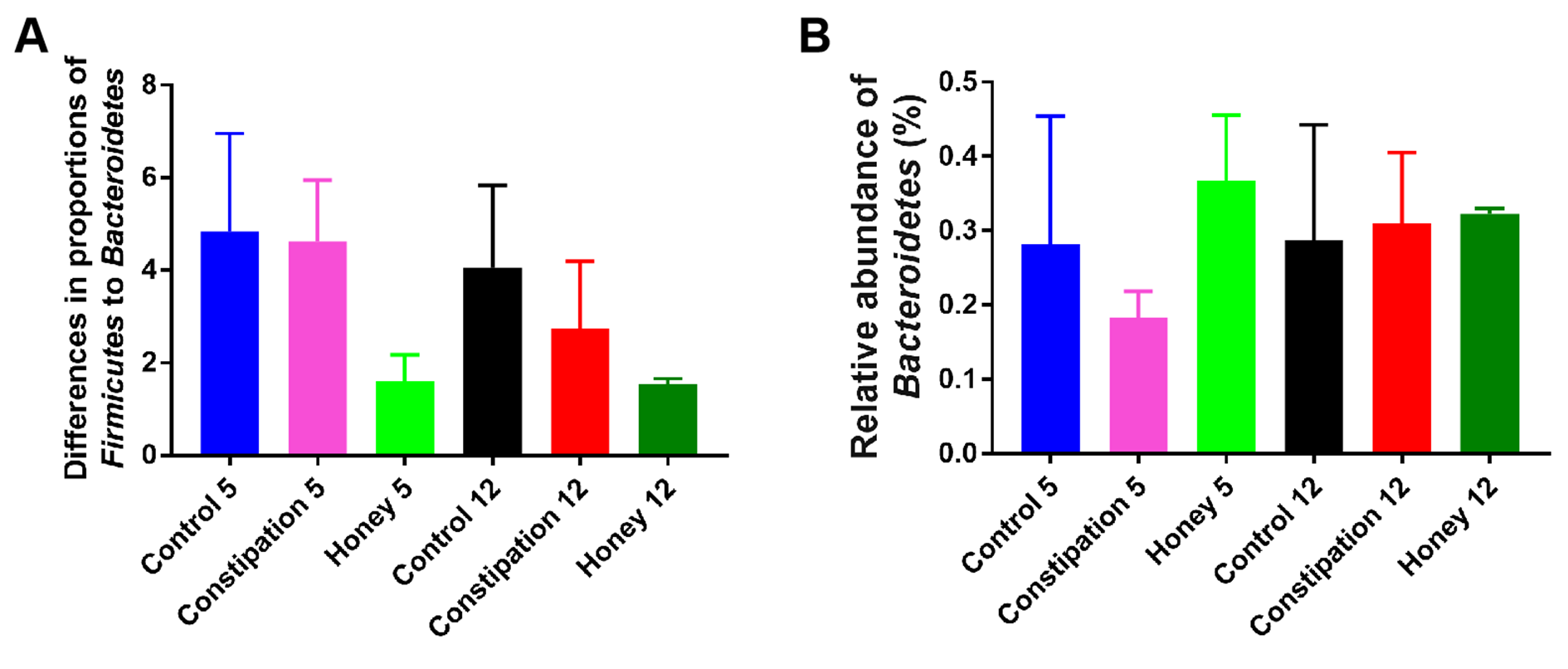

Supplement: Supplementary file 1 — Fig S1 [file FSN3-8-4388-s001.tif]
